# Vacuum–Laser Fabrication of Programmable Soft Actuators

**DOI:** 10.1002/advs.202522500

**Published:** 2026-03-08

**Authors:** Ashkan Rezanejad, Mostafa Mousa, Yuxuan Wang, Michael Adlerstein, Junke Yao, Antonio E. Forte

**Affiliations:** ^1^ Department of Engineering Science University of Oxford London UK; ^2^ Department of Engineering King's College London London UK

**Keywords:** bending actuators, laser cutting, plastic pouches, soft robotics, vacuum

## Abstract

Soft robotic actuators enable lightweight and compliant motion, but their fabrication typically relies on silicone molding, 3D printing, or textile lamination—processes that require expensive materials, long production times, or complex fabrication protocols. We introduce a rapid manufacturing strategy using low‐cost thermoplastic pouches that combines vacuum processing and laser cutting. By removing air gaps between layers, this method enables precise sealing and cutting, allowing complex inflatable geometries to be fabricated in under 10 min at a material cost below $0.10 per actuator. Compared to silicone elastomers, the reduced compliance of thermoplastics minimizes deformation losses and channels more energy into effective stiffening. The reliability of the method is verified through material testing and repeatable pressurization experiments, including response times of approximately 0.4s at operating pressure of 50–70kPa. We further use finite element modeling to predict bending behavior, derive geometric rules for programmable deformation, and construct a surrogate model for inverse design of homogeneous and heterogeneous bending actuators. Using this framework, target shapes such as alphabetic letters and spirals are achieved, and functional soft robotic prototypes, including crawlers, swimmers, and soft grippers, are demonstrated. These results position vacuum–laser processing as an accessible and scalable platform for rapid fabrication of adaptive soft robotic systems.

## Graphical abstract

A rapid and accessible fabrication strategy for inflatable soft actuators is presented, combining vacuum sealing with laser cutting of low‐cost thermoplastic pouches. The method enables precise sealing, fast fabrication, and programmable multi‐cell geometries. Integrated modeling and inverse design guide actuator performance, enabling robust bending actuators and functional soft robotic systems.

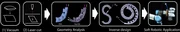

## 1 Introduction

Soft robotics uses compliant materials that deform under load, enabling safe interaction with humans and delicate environments [1, 2, 3]. Their inherent flexibility allows soft robots to adapt to unstructured or hazardous terrains where rigid robots struggle [4, 5, 6]. This compliance also supports dexterous tasks like grasping fragile objects or fine manipulation [7, 8, 9]. As a result, soft robots are well‐suited for biomedical devices [10], wearables [11], search‐and‐rescue [4], and autonomous exploration [5]. Central to these systems are soft actuators, which convert inputs such as pressure, heat, or electrical energy into mechanical motion [10, 11]. Beyond pneumatic systems, soft actuators based on alternative transduction mechanisms have also been explored at the device level, including moisture‐responsive composite actuators [12].

Among the different types of socift actuators, pneumatic bending actuators represent one of the most widely studied and versatile designs [13]. Inspired by natural organisms, bending actuators can achieve basic locomotion and manipulation modes such as curling, gripping, and crawling [5, 7, 14]. A well‐known example is the PneuNet actuator, which consists of a series of interconnected chambers that expand asymmetrically when pressurized, producing a bending deformation due to the presence of a strain‐limiting layer on one side [15]. Variants of this design have been employed to create multifunctional robots, including grippers capable of handling fragile objects, locomotion systems for soft crawlers, and continuum manipulators [5, 8, 16].

Conventional fabrication methods of soft actuators typically rely on casting silicone elastomers into molds [8], 3D printing complex chamber geometries [17], or laminating textiles with airtight membranes [18]. While effective, these approaches often require specialized equipment, prolonged processing times, and relatively expensive consumables. For instance, silicone casting demands custom mold preparation and curing, which slows prototyping cycles and limits scalability. 3D printing enables more intricate geometries but is constrained by printing resolution, material compatibility, and high equipment costs. Textile lamination, on the other hand, can provide lightweight and flexible actuators but often involves multiple processing steps and limited control over chamber geometry [18, 19]. These barriers hinder the rapid prototyping and deployment of soft robotic systems, particularly in settings where affordability and accessibility are critical.

An alternative pathway for soft actuator fabrication involves the use of fabrics or thermoplastic pouches. Thermoplastic‐coated textiles, such as nylon coated with thermoplastic polyurethane (TPU), have been widely employed due to their durability, flexibility, and ability to form airtight seams through heat pressing [20, 21, 22, 23, 24]. Recent work has demonstrated the fabrication of inflatable kirigami actuators by laser cutting TPU‐coated textiles and inserting a sacrificial paper mask before heat pressing, which prevents bonding in selected regions and defines the inflatable channels [25]. These materials allow for the creation of inflatable structures that are mechanically robust and suitable for repeated actuation. Recent research has demonstrated their versatility, with TPU‐coated fabrics enabling encoded soft‐textile robots [26], programmable fluidic devices [23], adaptive garments [27], and inflatable thermal actuators [28]. Similarly, thermoplastic pouches represent an appealing option because they are inexpensive, lightweight, and readily available. When appropriately sealed, they can hold pressure and deform into programmed shapes, enabling actuation without the need for complex fabrication steps. Prior studies highlight the ability to generate complex curvilinear motion under inflation [29] and the potential for scalable, automated fabrication through heat‐sealing methods [23], ultrasonic welder [30] or a laser cutter [24, 25]. However, despite their potential, they often rely on custom‐built heat‐sealing rigs to achieve more complex geometries, which limits their accessibility and scalability.

In this work, we introduce a new manufacturing strategy for thermoplastic pouch actuators that combines vacuum processing and laser cutting. The method begins by vacuum‐sealing thermoplastic bags to eliminate air gaps between layers, thereby creating a flat and uniform substrate. These pouches are then processed using a laser cutter, which is capable of both sealing and cutting the material with high precision. By carefully tuning laser power and speed, it is possible to generate airtight seals that define the geometry of inflatable chambers alongside through‐cuts that create openings. This dual functionality enables the fabrication of complex geometries directly from flat pouches in a matter of minutes.

The proposed method offers several key advantages over existing techniques. It is low cost, as the consumable materials (commercial thermoplastic bags) cost less than $0.10 per unit, a vacuum machine can be obtained for under $30, and a low‐power laser cutter (engraver) can be purchased for less than $500. It is also fast and scalable, with a typical actuator fabricated in less than 10 min, making it well suited for rapid prototyping and iteration. Importantly, the fabrication pipeline is simple and accessible, relying exclusively on commercially available equipment and requiring no custom‐built machinery, molds, or heat‐sealing rigs. Finally, it is versatile: the same approach can be used to create a wide range of inflatable geometries, and the reduced compliance of thermoplastics compared to silicone elastomers minimizes wasted deformation while channeling more energy into useful stiffening, thereby improving actuation efficiency. This effect is further supported by comparative force‐efficiency measurements against silicone pouches reported in Section S13.

Building on this foundation, we establish design principles for programmable bending through multi‐cell architectures, supported by finite element simulations and validated experimentally. We further develop a surrogate model that enables inverse design of both homogeneous and heterogeneous actuators, which we use to achieve complex target geometries such as alphabet letters and spirals. Lastly, we demonstrate the versatility of the approach through soft robotic applications including crawlers, swimmers, and a soft gripper. Overall, these results demonstrate that vacuum‐laser processing offers an accessible, efficient, and scalable route toward the next generation of adaptive, multifunctional soft robotic systems

## 2 Results

### 2.1 Manufacturing Technique

Here, we introduce a new manufacturing method for cutting and sealing thermoplastic pouches (commercial PA/PE nylon–polyethylene laminates) to create geometrically complex inflatables, bending actuators, and soft robots. The process begins by vacuuming thermoplastic bags, which eliminates the air gap between their two layers. This allows the material to be sealed and cut using a laser cutting device.

**FIGURE 1:**
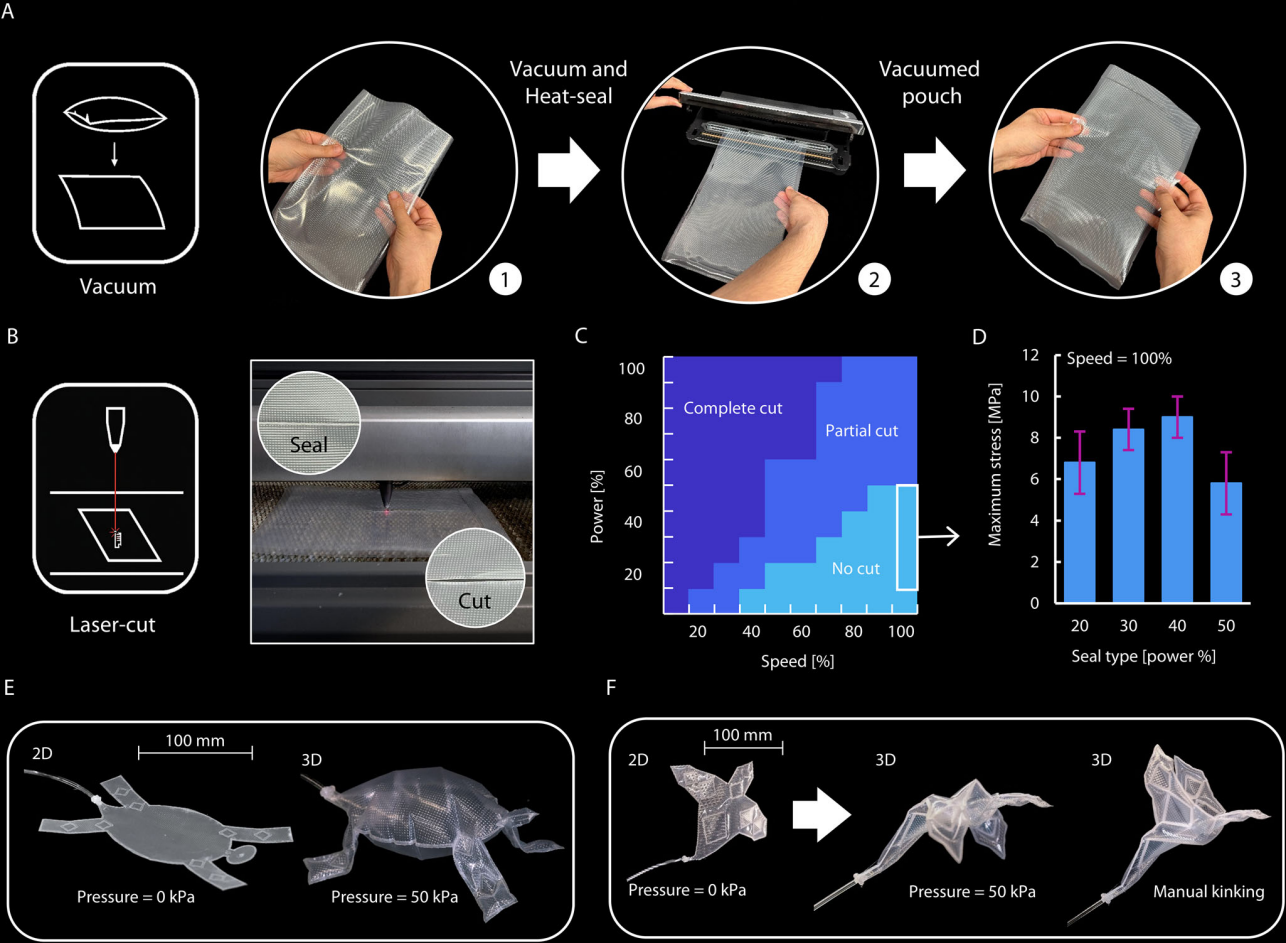
Manufacturing process of inflatable actuators using laser cutting. (A) Vacuuming thermoplastic pouches using a commercial vacuum and heat‐sealing machine. (B) Laser cutting thermoplastic pouches to produce cuts and seals. (C) Phase diagram showing the effect of laser cutter speed and power on the cut type of vacuumed pouches. (D) Mean and standard deviation of the maximum stress endured by the pouch seal before rupture in a tensile strength test. (E) An inflatable turtle with bistable legs that kink out of plane. (F) An inflatable bird with bistable wings, demonstrating reconfiguration of its flight posture while inflated.

We start with a thermoplastic bag sealed on three sides and open on one side, with the aim of removing the air completely before sealing the bag. We use a commercially available vacuum sealer (Bonsenkitchen Vacuum Sealer, Amazon.co.uk), which requires approximately ten seconds to draw a full vacuum and seal the thermoplastic bag (Figure 1A). Additionally, we develop an alternative method using a custom‐designed 3D‐printed outlet adaptor, enabling air removal and sealing when a vacuum sealer is not available (for more details, refer to Section S1).

After obtaining a fully sealed and vacuumed thermoplastic pouch, we use a laser cutter (VLS3.60DT, Universal Laser Systems) to both produce cuts and form sealed bonds between the two layers of the thermoplastic pouches (see Figure 1B).

By varying the laser speed and power from 10% to 100%, while fixing the z‐axis position at 0.5 mm, we observe three distinct outcomes (Figure 1C): i) complete cut, where the thermoplastic is fully severed and an opening is formed; ii) partial cut, where only portions are opened while other areas remain fused; and iii) no cut, where the layers remain intact and the laser instead forms a sealed bond between them. Notably, in the “no cut” regime, the laser energy is sufficient to fuse the two layers without penetrating the material; a process that we hereafter refer to as sealing.

To minimize the processing time required for sealing, we examine conditions at 100% speed and find that powers greater than or equal to 20% consistently produce a seal by heat‐bonding the two layers of the pouch. The strength of these seals is evaluated using a tensile testing machine (Instron). The results, presented in Figure 1D, show the maximum stress (force divided by the cross‐sectional area) that each seal can withstand before rupture. Each condition is tested five times, and standard deviations are reported as error bars. Among the tested conditions, the seal formed at 40% power exhibits the highest maximum withstanding stress ∼ 9 MPa (see Figure S2C for the image of the seal‐strength test setup). Based on these results, we adopt a speed of 100% and a power setting of 40% for all subsequent pouch sealing operations throughout the manuscript. Additional characterization of pouch bursting pressure and inflation response time is provided in Section S3.

To demonstrate that this manufacturing technique enables inflatable actuators with complex geometries, we fabricate two artistic structures. First, we design an inflatable turtle whose legs kink out of plane, forming a 3D structure (Figure 1E). We also design an inflatable bird (design inspired by [22]) that leverages kinking to enable manual reconfiguration of its shape while inflated, allowing changes in its flight posture (Figure 1F). Both inflatables are pressurized to an internal pressure of 50 kPa (for design sketches, refer to Section S4.)

### 2.2 Multi‐Cell Design Approach for In‐Plane Bending Actuators

#### 2.2.1 The Design Principles

**FIGURE 2:**
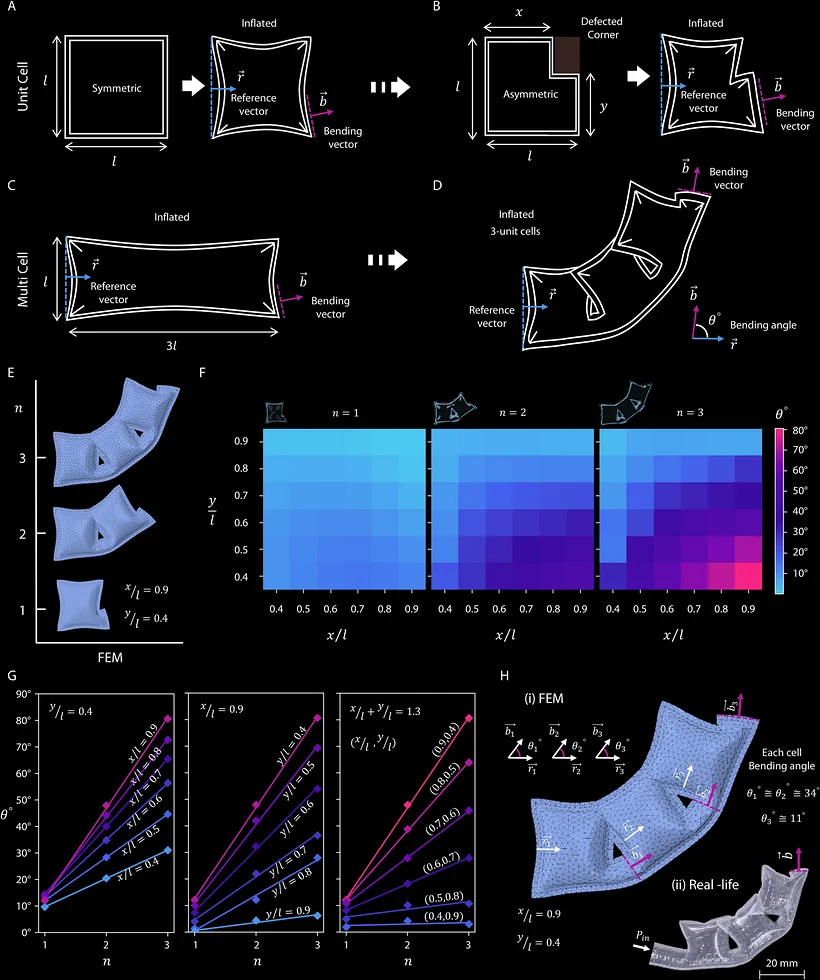
Multi‐cell design principles and finite element analysis. (A) Symmetric unit cell with edge length *l*, shown uninflated (left) and inflated (right). (B) Asymmetric unit cell with a defected corner defined by two new geometric parameters, *x* and *y*, uninflated (left) and inflated (right). (C) Inflated multi‐cell structure consisting of three connected symmetric unit cells. (D) Inflated multi‐cell structure consisting of three asymmetric unit cells. (E) FEM‐simulated structures with varying number of cells *n* = [1, 2, 3]. (F) Grid‐search heatmap of bending angles *θ* for simulated actuators with varying geometric parameters (*x/l, y/l*) and number of cells *n*. (G) Linear relationship between bending angle *θ* and number of cells *n* for cases with fixed geometric features (*x/l, y/l*). (H) (i) Bending behavior of individual unit cells in a multi‐cell structure. (ii) Real‐life prototype with the same geometric features.

Using our new manufacturing technique, we can fabricate a symmetric square‐shaped pouch with edge length *l*. Upon inflation, this pouch transforms into a cushion‐like geometry with all four sides curving inward. To analyze its deformation, we draw an imaginary line from one vertex of the square to the midpoint of its adjacent edge and compute the normal vector to this line. We define this normal as the bending vector. During inflation, the bending vector changes its angle relative to a reference vector, which we define as the normal to another imaginary line connecting the two opposite vertices of the square. This angular change introduces a slight bending (see Figure 2A). Force output and cyclic durability measurements of square pouches are reported in Section S5.

If we treat the square pouch as a unit cell and extend one of its edges threefold (edge length = 3*l*) to build a multi‐cell, the bending vector maintains its direction upon inflation, providing no control over bending (see Figure 2C). To address this limitation, we introduce a defected corner into the unit‐cell design, creating an asymmetric geometry characterized by two new parameters, *x* and *y* (see Figure 2B). This shape resembles previous and well‐known soft actuator designs [15, 24]. When three of these asymmetric unit cells are connected to form a multi‐cell, the bending vector can shift significantly relative to the reference vector. We denote this angular deviation as the bending angle *θ* (see Figure 2D).

### 2.3 Geometric Analysis

We simulate the inflation of the designed cell using Finite Elements (FEM, Abaqus, for Method details refer to Section S6), varying the aspect ratios *x/l* and *y/l* within the range [0.4, 0.9] to measure the bending angle *θ* across the design space. We repeat this analysis for three structures composed of homogeneous cells (*n* = 1, 2, 3; see Figure 2E). The resulting grid search (Figure 2F) shows that increasing the number of cells produces higher bending angles. The geometric parameters also strongly influence *θ*: larger *x/l* and smaller *y/l* yield higher bending, reaching nearly 80*^◦^* for the multi‐cell with *n* = 3, *x/l* = 0.9, and *y/l* = 0.4.

We further observe a linear relationship between the bending angle *θ* and the number of cells *n* when the geometric features (*x/l, y/l*) are fixed. We demonstrate this linearity by plotting *θ* against *n* for multiple design cases extracted from the heatmap: the bottom row (*y/l* = 0.4), the rightmost column (*x/l* = 0.9), and the reverse diagonal (*x/l* + *y/l* = 1.3) shown in Figure 2G.

This linear trend holds for *n >* 1; each design starts with an offset. To explain this behavior, we examine the bending angles of individual cells in a multi‐cell configuration (*n* = 3*, x/l* = 0.9*, y/l* = 0.4). The first two cells bend almost equally (*θ*_1_ ≈ *θ*_2_), while the third cell bends significantly less (*θ*_3_). Interestingly, *θ*_3_ matches the bending angle of a single isolated cell (*n* = 1) with the same geometric features. This indicates that the last cell, which is not connected to a subsequent cell, defines the offset in the bending angle of multi‐cell structures (Figure 2H–i).

Finally, we validate the simulations experimentally by fabricating a three‐cell actuator (*n* = 3*, x/l* = 0.9*, y/l* = 0.4) with an inlet pressure of *P*_in_ = 50 kPa. The measured bending angle of 82*^◦^* closely matches the FEM prediction of 80*^◦^* (Figure 2H–ii).

### 2.4 Inverse Design Surrogate Model

After obtaining the dataset from the geometric analysis performed in FEM, our goal is to establish a functional relationship between the unit‐cell geometry (*x/l, y/l*), the number of cells *n*, and the resulting bending angle *θ*. The analysis takes place at a fixed pressure of 50 kPa, yielding data points of the form:

(1) $$\left(x/l,\;y/l,\;n\right)\to \theta$$

Since FEM simulations are computationally expensive and were performed only up to *n* = 3, a surrogate model is required to estimate the bending response for larger *n*. As the bending angle exhibits an approximately linear dependence on the number of cells *n* for a given (*x/l, y/l*) (see Figure 2G), a surrogate model will likely be reliable when extrapolating to larger *n* values beyond those available from FEM.

We employ a regression model to fit the FEM data using a quadratic polynomial via the least‐squares method (see Section S7 of the Supporting Information for details). The resulting polynomial achieves an *R* ^2^ score of 0.95, allowing it to serve as a surrogate model for the inverse design problem: given a desired target bending angle θ_*t*_, the model predicts the bending angle $\hat{\theta }$ for each combination of *n* and (*x/l, y/l*), enabling us to identify which geometries best achieve the target:

(2) $$\theta_{t}\to \hat{\theta }\left(x/l,\;y/l,\;n\right)$$

In this work, we consider *n* ∈ {1*,…*, 10}, and for each *n* we select the candidate geometry (*x/l, y/l*) whose predicted bending angle $\hat{\theta }$ is closest to the target θ_*t*_. A design is considered eligible only if the bending angle prediction falls within ±10% of the target:

(3) $$\theta_{t}\left(1-0.1\right)\leq \hat{\theta }\left(x/l,\;y/l,\;n\right)\leq \theta_{t}\left(1+0.1\right)$$

**FIGURE 3:**
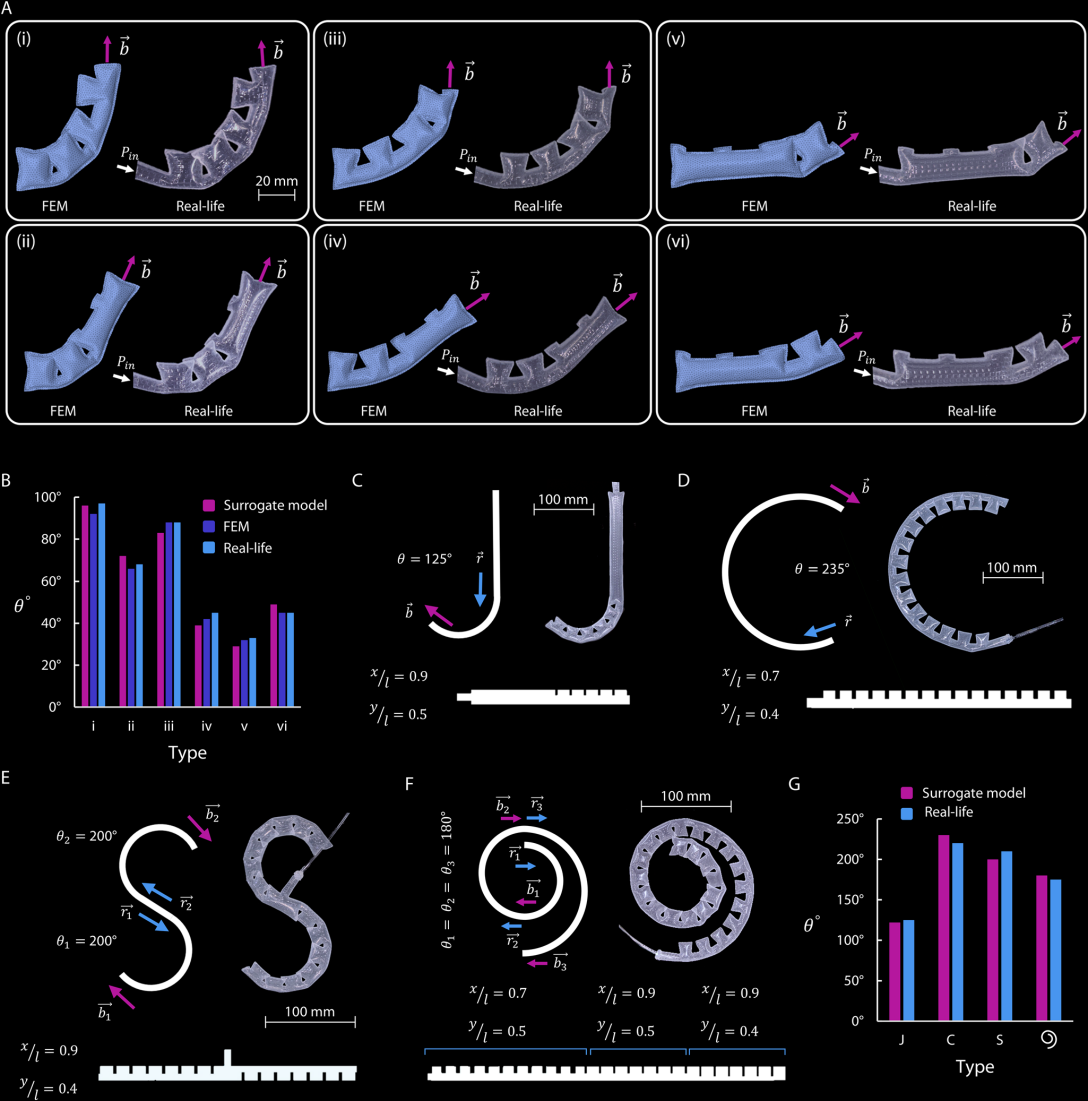
Heterogeneous multi‐cell design and inverse design of complex geometries. (A) Heterogeneous multi‐cells with *n* = 4, constructed from six combinations of homogeneous multi‐cells with *n* = 2, shown as FEM simulations (left) and real‐life prototypes (right). (B) Bending angle *θ* for each heterogeneous type as predicted by the surrogate model, FEM, and measurements of real‐life prototypes. (C) Inverse design of the letter “J” using the surrogate model and prototype, consisting of a homogeneous multi‐cell capable of 125*^◦^* bending. (D) Inverse design of the letter “C” using the surrogate model and prototype, consisting of a homogeneous multi‐cell capable of 235*^◦^* bending. (E) Inverse design of the letter “S” using the surrogate model and prototype, consisting of a heterogeneous multi‐cell formed by two homogeneous multi‐cells oriented in opposite directions, each capable of 200*^◦^* bending. (F) Inverse design of a spiral geometry using the surrogate model and prototype, consisting of a heterogeneous multi‐cell of three connected homogeneous multi‐cells with different geometric features, each capable of 180*^◦^* bending and varying in arc length. (G) Comparison of bending angles *θ* between surrogate model predictions and prototype measurements.

If no (*x/l, y/l*) pair satisfies this tolerance for a given *n*, then the target bending angle is considered not achievable for that *n*. However, there may be multiple values of *n* that can achieve the desired bending angle. This is especially useful when inverse‐designing a bending structure composed of multiple bending segments that share the same bending angle but differ in length, such as a spiral structure (see Figure 3F). Overall, this model not only identifies a structure that satisfies the required bending, but also provides flexibility in choosing the actuator length by selecting *n*.

### 2.5 Heterogeneous Multi‐Cells

So far, we have studied homogeneous multi‐cells and predicted their bending angle from the linear relationship with the number of cells *n*. However, we now investigate the bending of heterogeneous multi‐cells with *n* = 4, constructed by combining two homogeneous multi‐cells of *n* = 2 but with different (*x/l, y/l*) parameters. This is to verify that the coupling between cells with different geometrical features is minimal.

We select three homogeneous types that exhibit high, medium, and low bending, with (*x/l, y/l*) features of (0.9, 0.4), (0.7, 0.6), and (0.4, 0.9), respectively. From these, we construct six heterogeneous multi‐cell types by pairing all possible combinations of the three, and we also fabricate real‐life prototypes of each design for comparison (see Figure 3A).

For heterogeneous multi‐cell structures, our surrogate model evaluates the bending of the two constituent homogeneous substructures. The first multi‐cell contributes without a bending offset, while the second introduces an offset due to the effect described earlier, where the last unit cell has a lower bending angle (Figure 2H–i). We report the bending angles of all six heterogeneous types as predicted by the surrogate model, FEM simulations, and experimental measurements (Figure 3B). While the results show strong agreement across the three methods, small deviations between the surrogate model, FEM predictions, and experimental measurements are observed (Figure 3B). These arise primarily from modeling simplifications, as the FEM does not explicitly capture viscoelastic effects or local wrinkling, which is dependent on mesh resolution (see Section S6 for details of FEM). Additionally, as the surrogate model is evaluated within a ±10% prediction tolerance, deviations of comparable magnitude with respect to experimental measurements are expected.

Furthermore, we employ the surrogate model to inverse‐design complex bending structures, including the English alphabet letters J (Figure 3C), C (Figure 3D), and S (Figure 3E). We also inverse‐design a more intricate geometry: a spiral composed of three 180*^◦^* arcs with varying arc lengths (Figure 3F). This design is achieved by selecting three multi‐cell structures that each enable 180*^◦^* bending but differ in the number of cells *n*, which determines the arc length. The results show strong agreement between the bending angles predicted by the surrogate model and those measured from the fabricated prototypes across all four complex geometries (Figure 3G).

Lastly, to assess the practical reliability and manufacturability of the proposed bending actuators, we evaluate their cyclic durability under repeated pressurization in Section S8 and investigate the scaling limits and performance under geometric up‐scaling and down‐scaling in Section S9.

### 2.6 Soft Robotic Applications

To demonstrate the versatility of the proposed bending actuator, we integrate it into several soft robotic systems that leverage its large bending angles and lightweight design. These examples highlight how simple geometric tuning enables a wide range of functionalities, from load lifting and grasping to terrestrial and aquatic locomotion.

#### 2.6.1 Stacked Benders

**FIGURE 4:**
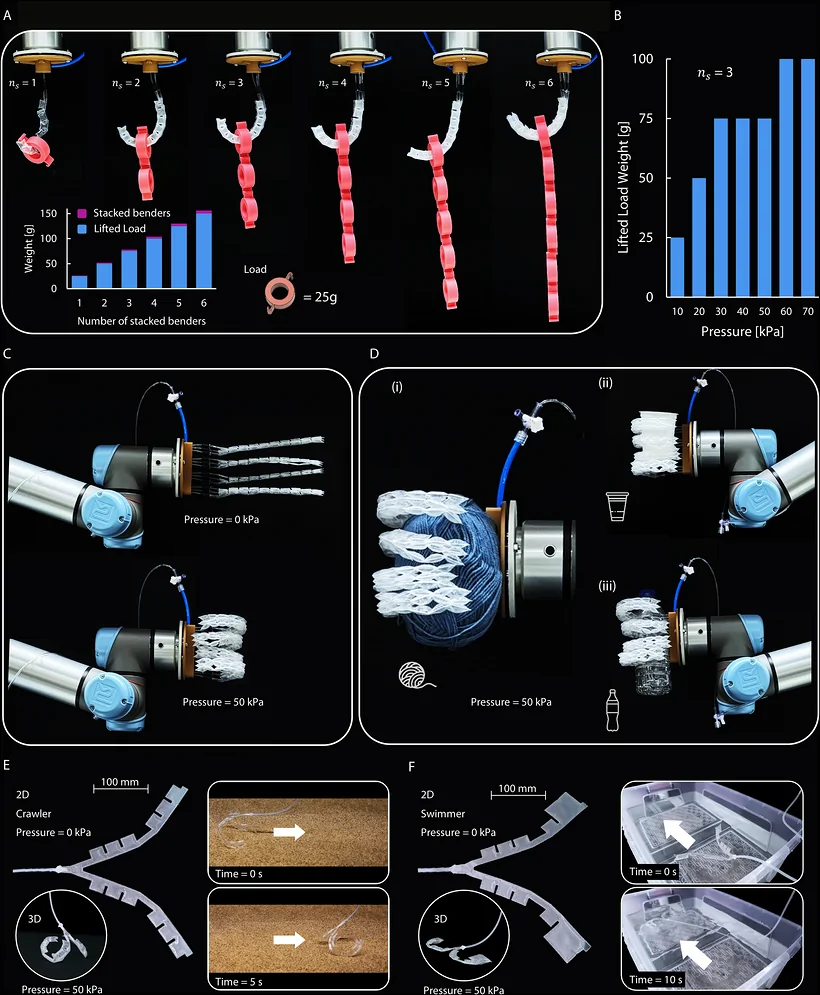
Stacked benders, soft robotic gripper, and locomotion. (A) Load‐lifting performance of stacked benders with varying numbers of layers (*n_s_* = [1*,…*, 6]) at an internal pressure of 50 kPa. (B) Effect of internal pressure on the lifting capacity of a three‐layer stacked bender (*n_s_* = 3). (C) A soft gripper composed of four stacked benders (*n_s_* = 3 each) arranged in an array with 20 mm spacing. (D) Demonstration of the soft gripper successfully lifting (i) a ball of yarn, (ii) a plastic cup, and (iii) a plastic bottle. (E) A soft crawler with two 30*^◦^* offset legs, actuated at 1.8 Hz and 50 kPa, achieving a speed of 0.8 body lengths per second. (F) A soft swimmer variant with paddle‐shaped legs, actuated at 1.2 Hz and 50 kPa, achieving a speed of 0.5 body lengths per second.

Using an adhesive (Instant Adhesive, Loctite), we bond identical bending actuators of one geometric type (*x/l* = 0.9*, y/l* = 0.4*, n* = 10) on top of each other to form stacked structures with *n_s_* = [1*,…*, 6], where *n_s_* denotes the number of actuators in the stack. Each stacked bender is tested at an internal pressure of 50 kPa to evaluate its load‐lifting capability. Custom 3D‐printed ring‐shaped weights, each weighing 25 g, are incrementally added to the tip of the actuator until it can no longer sustain the load and buckles, causing the weights to fall. The maximum stable load, the heaviest load lifted without failure, is recorded as the lifted weight. The results show a linear relationship between *n_s_* and the lifted load: as the number of stacked benders increases, the total lifting capacity increases proportionally. Each individual bender weighs approximately 1 g and can lift about 25 times its own weight when inflated. This trend holds across all tested configurations, for example, a stack with *n_s_* = 6 (≈ 6*g* total mass) can lift a 150 g load (Figure 4A). Lastly, we examine how internal pressure influences the load‐lifting performance of the stacked benders. For the configuration with *n_s_* = 3, the lifting capacity increases with pressure, demonstrating that higher internal pressures yield greater output force (Figure 4B). Additionally, we demonstrate that selective actuation of stacked bending actuators can also enable out‐of‐plane bending modes (Section S10).

#### 2.6.2 Soft Robotic Gripper

Moreover, we design a soft gripper composed of four stacked benders, each with a configuration of *n_s_* = 3, arranged in an array with a 20 mm spacing between adjacent benders. When inflated to 50 kPa, the benders bend by approximately 320*^◦^*, forming an effective gripping mechanism (Figure 4C). The gripper is mounted on a UR5e robotic arm (Universal Robots) and is able to successfully pick up three lightweight objects (each *<* 200 g): a ball of yarn (Figure 4D–i), a plastic cup (Figure 4D–ii), and a plastic bottle (Figure 4D–iii). The gripper achieves a success rate of 100% in grasping and lifting these objects, provided they are positioned within its reach. Additional robustness tests on different target shapes and measurements of gripping force are reported in Section S11.

#### 2.6.3 Soft Crawler and Swimmer

Furthermore, we develop an ultralight (*<* 5 g) fully soft crawler. The crawler design consists of two legs separated by a 30*^◦^* angle, with each leg comprising two sets of three unit cells. The two sets are connected at an additional offset angle of 30*^◦^*. A crease is impressed along the central axis of symmetry to bias the structure toward out‐of‐plane deformation, enabling the crawler to morph into a 3D shape upon pressurization, with its legs bending beyond 180*^◦^*. A Microfluidic Flow Controller (OB1 MK4, Elveflow) is used to generate an oscillation frequency of 1.8 Hz at a pressure of 50 kPa. Owing to its lightweight design, the crawler achieves a speed of 0.8 body lengths per second (Figure 4E).

Additionally, we modify the crawler design by replacing two of the end unit cells on each leg with a single larger unit cell, creating a paddle shape to develop a swimmer. Using the same Microfluidic Flow Controller (OB1 MK4, Elveflow), we generate a lower oscillation frequency of 1.2 Hz at a pressure of 50 kPa. The swimmer achieves a speed of 0.5 body lengths per second (Figure 4F). For design sketches of the crawler and the swimmer, refer to Section S12. Additionally, the effect of actuation frequency on locomotion speed for both the crawler and swimmer is systematically characterized in Section S12.

## 3 Conclusion

In this work, we present a rapid and low‐cost manufacturing strategy for thermoplastic pouch actuators that combines vacuum sealing with laser cutting. We establish its reliability through material testing of laser‐sealed pouches and demonstrate its effectiveness by fabricating a wide range of inflatable actuators. Beyond manufacturing, we introduce a design framework for programmable bending based on multi‐cell architectures. Finite element modeling serves as a predictive tool to explore the effect of geometric parameters and cell number on bending behavior, while a surrogate model captures these relationships and enables inverse design. Using this approach, we build both homogeneous and heterogeneous actuators, achieving complex geometries such as letters and spirals that closely match model predictions.

Compared to existing inflatable actuator fabrication approaches based on heat sealing, ultrasonic welding, or laser processing [23, 24, 25, 30], the proposed method operates in a distinct and complementary regime that emphasizes rapid fabrication, quantitative performance reporting, and accessibility. Using commercially available vacuum sealers and laser cutters, actuators are fabricated in under 10 min from inexpensive thermoplastic pouches (PA/PE, material cost less than $0.10 per unit). In contrast, fabrication time and reproducibility are often not explicitly reported in related studies, which typically rely on multi‐step or manually intensive processes. Although PA/PE pouches are inexpensive and widely available, they are difficult to process using conventional heat‐press methods (commonly employed as an initial step in laser‐cut fabrication workflows) due to their temperature sensitivity and narrow sealing window; the proposed vacuum–laser approach bypasses this step, eliminates sacrificial layers, reduces fabrication complexity, and substantially shortens fabrication time.

From a mechanical perspective, vacuum–laser thermoplastic pouches achieve bulging forces of approximately 202 N at 50 kPa, corresponding to a normalized output force of approximately 0.033 N/mm^2^, alongside vertical output forces of approximately 4.5 N (Section S5). By comparison, silicone pouches of similar dimensions (in particular same thickness) fabricated from a commonly used elastomer (Ecoflex 30) rupture at approximately 4 kPa and exhibit a maximum bulging force of about 28 N, corresponding to a lower normalized output force of approximately 0.002 N/mm^2^ (Section S13). Reported blocking forces in related inflatable and textile‐based actuator systems are typically on the order of 0.1–3 N at comparable pressures [24, 30], or reach approximately 10–15 N only at substantially higher operating pressures exceeding 200 kPa [25]. In addition, we systematically characterize durability under up to 100,000 inflation–deflation cycles for both square pouches and bending actuators (Sections S5 and S8), highlighting the importance of reporting practical fabrication and lifetime metrics alongside geometric programmability.

Notably, this technique has been validated only with commercially available PA/PE pouches, selected for their accessibility as vacuum‐sealable materials; future studies may explore other vacuum‐processable and laser‐compatible thermoplastics, such as TPU‐coated nylon fabrics and polyethylene‐based films, with particular attention to material‐dependent laser parameters.

Lastly, we demonstrate the versatility of the vacuum‐laser fabrication method through a series of soft robotic prototypes, including crawlers, swimmers, and a soft gripper, all driven by programmable bending actuators. These results establish vacuum‐laser processing as a practical, accessible, and scalable manufacturing paradigm for inflatable soft robotics. The presented bending actuator framework, built on tunable multi‐cell geometries, provides a foundation for systematic control of deformation modes and can be extended to more complex actuation behaviors.

Further exploration of the geometric design space, including cell orientation, offset angles, and structural arrangements, may enable new actuation modes such as twisting or out‐of‐plane buckling. Optimization of these parameters could yield soft robots capable of amphibious locomotion, complex manipulation, or reconfigurable morphologies. In addition, expanding the soft gripper architecture by varying the number, distribution, and geometry of bending actuators relative to object mass could lead to enhanced dexterity and adaptability. Together, these directions highlight the broad potential of vacuum‐laser fabricated bending actuators as a platform for multifunctional, programmable, and scalable soft robotic systems.

## 4 Experimental Section

Refer to the Supporting Information for additional details on the fabrication of vacuumed pouches, schematic designs of the inflatables and bending actuators, as well as descriptions of the surrogate model and supplementary experiments.

## Funding

The authors would like to acknowledge the financial support of UK Research and Innovation: UKRI grant MR/X035506/1 (A.E.F).

## Conflicts of Interest

The authors declare no conflicts of interest.

## Supporting information




**Supporting File 1**: advs74289‐sup‐0001‐SuppMat.pdf.

**Supporting File 2**: advs74289‐sup‐0002‐MovieS1.mp4.

## Data Availability

The data that support the findings of this study are available from the corresponding author upon reasonable request.
