# Supplementary material for: Vacuum–Laser Fabrication of Programmable Soft Actuators
Source: Adv Sci (Weinh). 2026 Mar 8;13(31):e22500. doi: 10.1002/advs.202522500 (PMC13588013; doi:10.1002/advs.202522500)
Supplement: Supplementary file 1 [file ADVS-13-e22500-s002.pdf]

Vacuum–Laser Fabrication of Programmable Soft Actuators

A. Rezanejad, M. Mousa, Y. Wang, M. Adlerstein, J. Yao, A.E. Forte

Supporting Information

S1 Custom Vacuum Adaptor Method for Air Extraction

In this approach (Fig. S1), we employ a custom-designed 3D-printed outlet adaptor. To attach the adaptor, a 6 mm hole is laser cut into one layer of the thermoplastic by placing a sheet of thick paper inside the bag to protect the opposite layer. The bottom part of the adaptor is inserted through the hole, and the top part (equipped with an O-ring to maximize the air seal) is screwed into place. After the adaptor is fitted, the open side of the bag is heat-sealed, and the bag is then fully evacuated using a vacuum generator (Festo Vacuum Generator, 8 bar, Farnell).

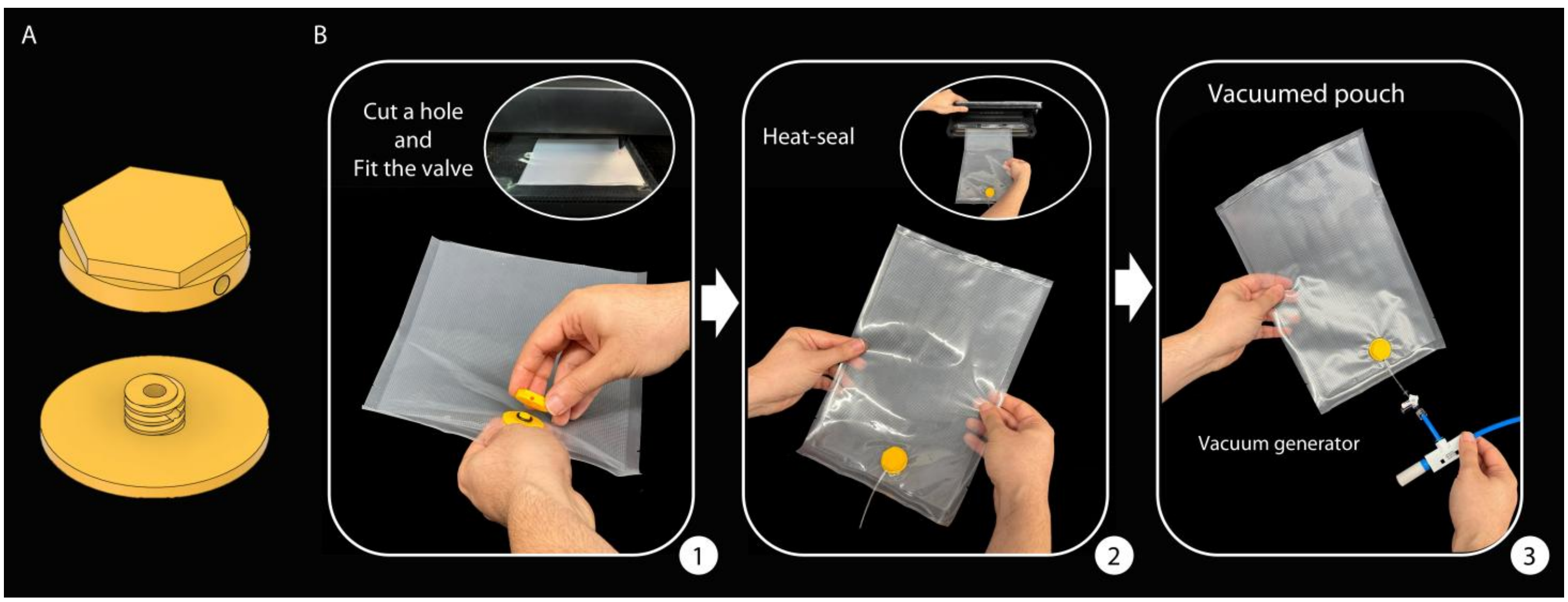

Figure S1: Custom Vacuum Adaptor Method for Air Extraction. (A) CAD design of the custom-made outlet. **(B)** Vacuuming thermoplastic pouches using a custom-made outlet, followed by heat sealing and air extraction with a vacuum generator.

S2 Tensile Testing

The mechanical response of the thermoplastic material (PA/PE nylon–polyethylene laminates) is characterized using uniaxial tensile testing (Instron). The specimen measured 50 mm in width, 100 mm in length, and 0.1 mm in thickness. From the measured force-displacement data, the stress-strain curve was obtained, as shown in Fig. S2 A.

A snapshot of the stretched specimen during tensile testing is presented in Fig. S1 B. An image of the seal-strength testing setup is shown in Fig. S2 C; The results are provided in Section 2.1 (see Fig. 1D).

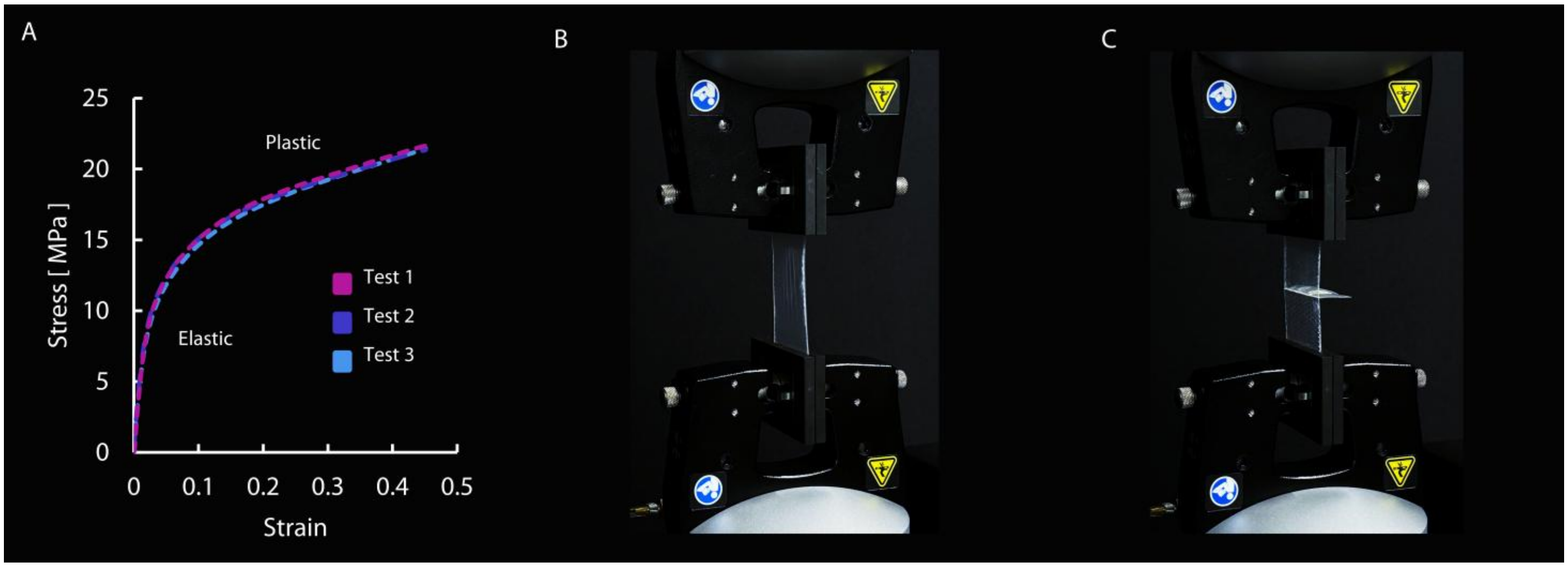

Figure S2: Tensile and Seal-Strength Testing. (A) Stress-strain curve of the thermoplastic material under uniaxial tension. **(B)** Snapshot of the tensile test performed on the thermoplastic specimen using the Instron mechanical testing system. **(C)** Seal-strength test of a laser-sealed thermoplastic specimen conducted using the Instron system.

S3 Bursting Pressure and Response Time of Vacuum-Laser Pouches

We design 100 mm by 100 mm square-shaped pouches with varying seal widths of [0.1, 0.2, 0.5, 1, 2, 5] mm to test the bursting pressure using a microfluidic pressure controller (OB1 MK4, Elveflow). The test is carried out by linearly increasing the input pressure until the pouch bursts or an opening is created that allows leakage. We monitor the pressure using a pressure sensor (ADP5150) controlled by a data acquisition module (National Instruments). We repeat the experiment for five samples per seal width and record the mean and standard deviation, as shown in Fig. S3A.

Seal widths below 0.2 mm fail to fully inflate and begin leaking before reaching an internal pressure of 10 kPa. A seal width of 0.5 mm exhibits a bursting pressure of approximately 50 kPa, whereas seal widths larger than 1 mm result in a consistent bursting pressure slightly above 70 kPa. For this reason, we choose a seal width of 1 mm for subsequent designs of inflatables and actuators, as it is the minimum seal width that achieves the maximum bursting pressure.

Furthermore, we characterize the response time of the same 100 mm by 100 mm square-shaped pouches under instantaneous pressure inputs applied using the microfluidic pressure controller. The response time is defined as the duration required for the pouch to transition from the uninflated state to full inflation at a given input pressure, which is measured from high-speed video recordings (DJI Action 4, 1080p, 240 fps). For each pressure condition, the experiment is repeated five times, and the corresponding mean and standard deviation are reported in Fig. S3B. For input pressures of 50 and 70 kPa, we measure a response time of approximately 0.4 s. Across all tested conditions, the standard deviation remains below 0.03 s, indicating highly repeatable material behavior enabled by our manufacturing technique.

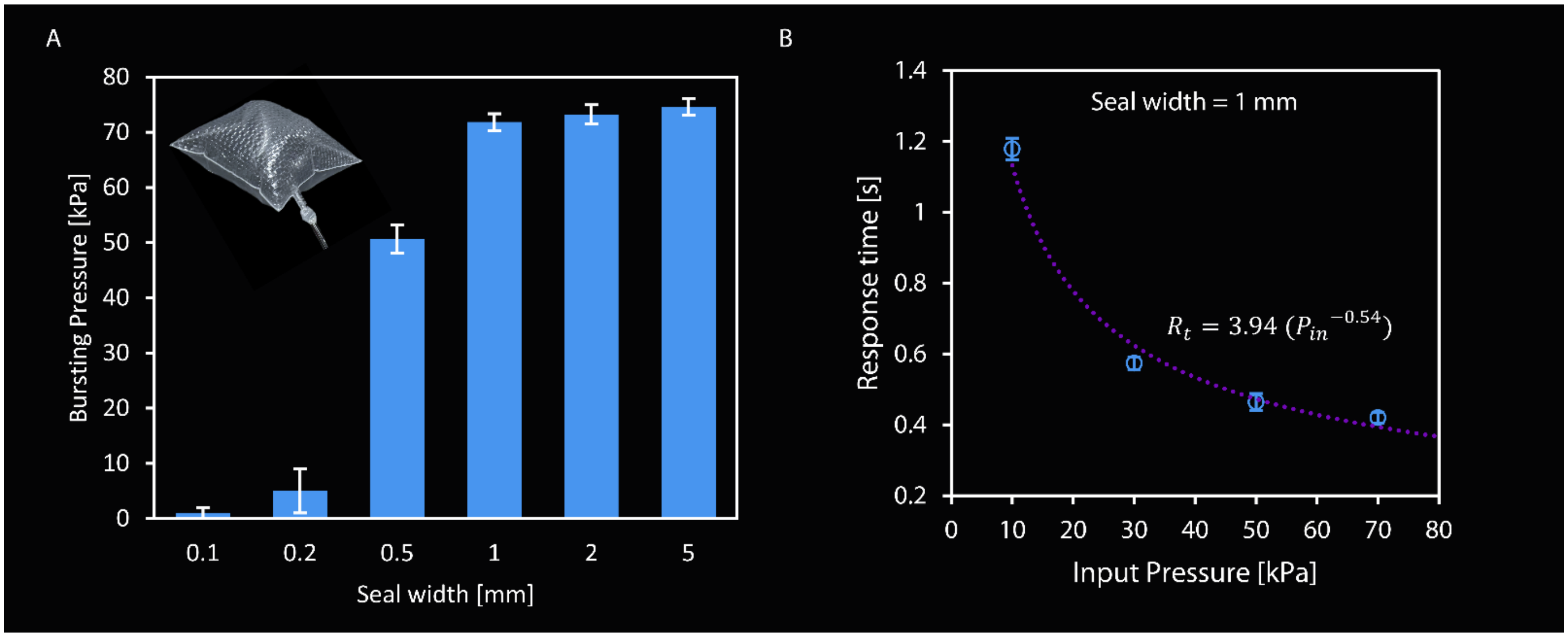

Figure S3: Vacuum-Laser pouches dynamic properties. (A) Bursting pressure of 100 mm × 100 mm pouches with varying seal widths. **(B)** Response time of the pouches to fully inflate under different input pressures.

S4 Inflatables Design Sketches

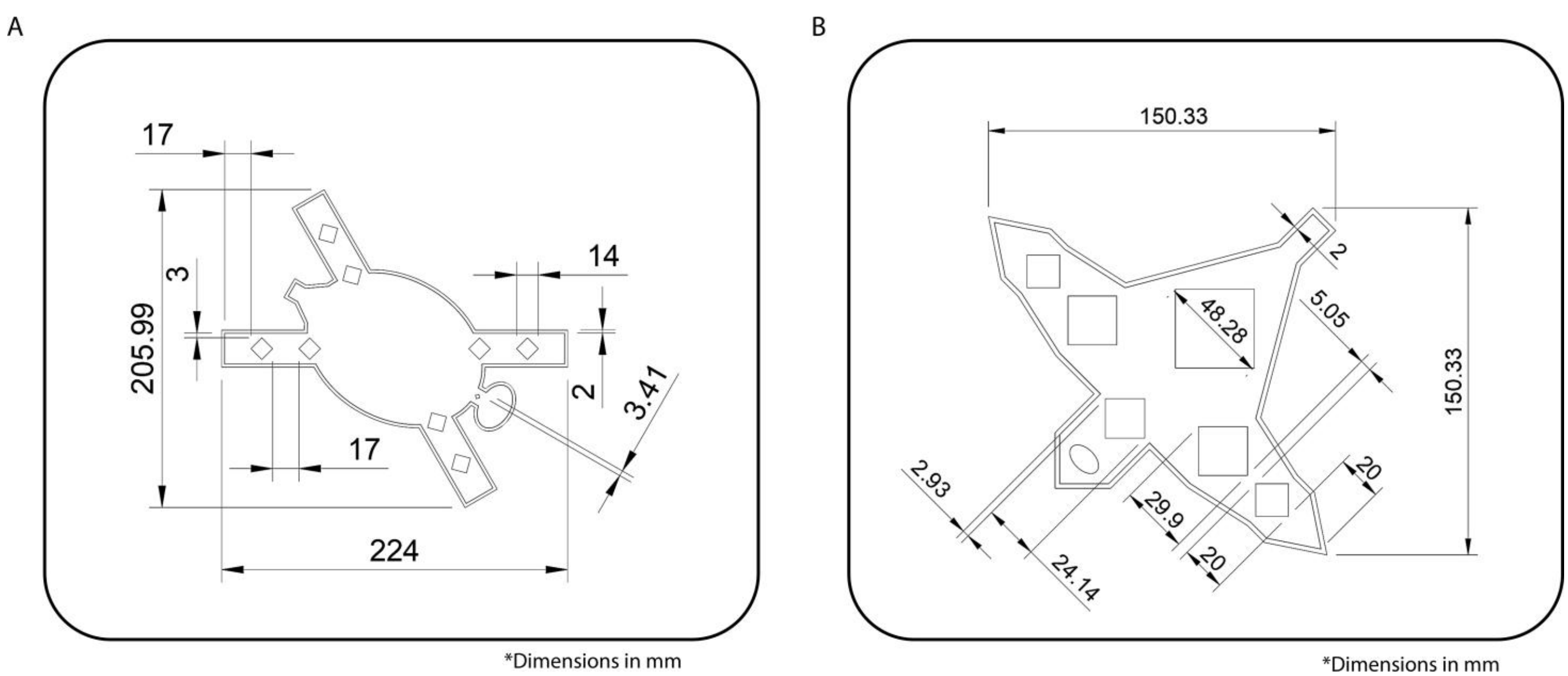

Figure S4: Inflatable turtle and bird technical drawings. (A) The turtle design sketch with 2 mm edges for sealing. **(B)** The bird design sketch with 2 mm edges for sealing.

S5 Force Output and Durability of Vacuum-Laser Pouches

We observe that upon inflation, a square-shaped pouch bulges and adopts an almost cushion-like shape. The midpoint of each edge moves inward, as illustrated in Fig. 2A. To investigate the linear force that this midpoint can withstand, we design a vertical force test in which a load is suspended from the midpoint of one edge of a square-shaped pouch (100 mm by 100 mm). The load rests on a laboratory digital scale and pulls the pouch downward. When pressure is applied to the pouch (using the microfluidic

pressure controller), the midpoint moves upward and partially lifts the load, as shown in Fig. S5A.

We intentionally select a sufficiently large load (7 N in this experiment) to ensure that the load is not fully lifted from the digital scale. This enables accurate measurement of the change in the recorded weight, which is then converted to force. The experiment is repeated using five pouch samples, and the mean vertical force and standard deviation are recorded for different input pressures, as shown in Fig. S5B. The maximum vertical force recorded at 50 kPa is approximately 4.5 N.

Furthermore, we design an experiment to evaluate the durability of the vacuum–laser pouches. Four pouches (100 mm by 100 mm) are connected to a microfluidic pressure controller (OB1 MK4, Elveflow; see Fig. S5C). The pressure controller is programmed to generate oscillating pressure with a 2 s cycle, switching between 0 and 50 kPa using a step function. The input pressure is measured using an external pressure sensor (ADP5150) controlled by a data acquisition module (National Instruments), and a 10 s sample of the pressure oscillations is reported in Fig. S5D. For the durability test, the pouches are subjected to 100,000 cycles of pressure oscillation between 0 and 50 kPa.

To compare the performance of newly fabricated vacuum–laser pouches with pouches that have undergone 100,000 cycles of oscillating pressure, we measure the bulging force. This force is measured by placing a pouch between two disks separated by 8 mm (this allows approximately 60% of the original area of the pouch to contact the top disk, 78 mm by 78 mm), allowing the pouch to bulge during inflation. The top disk is positioned directly below the tensile testing machine load cell, enabling measurement of the bulging force upon inflation (Instron; see Fig. S5E). We measure the bulging force of four newly fabricated pouches under different input pressures and repeat the same measurements for pouches subjected to 100,000 pressure cycles. The mean bulging force and standard deviation are reported in Fig. S5F.

The results show an approximately 5% reduction in bulging force for pouches that have undergone 100,000 cycles of oscillating pressure compared to newly fabricated pouches (approximately 202 N versus 192 N, respectively). This modest decrease indicates that the vacuum–laser pouches exhibit high durability and are able to retain about 95% of their original force output after 100,000 pressure cycles.

In addition, we measure the normalized bulging force of the pouches. This is obtained by dividing the measured force by the effective pouch area in contact with the top disk. Approximately 60% of the original pouch area is in contact with the disk, corresponding to a contact area of 78 mm×78 mm. Using this definition, we obtain normalized bulging forces of approximately [0.006, 0.013, 0.019, 0.026, 0.033] N/mm2 at input pressures of [10, 20, 30, 40, 50] kPa, respectively, at 1 cycle, and [0.006, 0.012, 0.019, 0.025, 0.031] N/mm2 after 100,000 cycles.

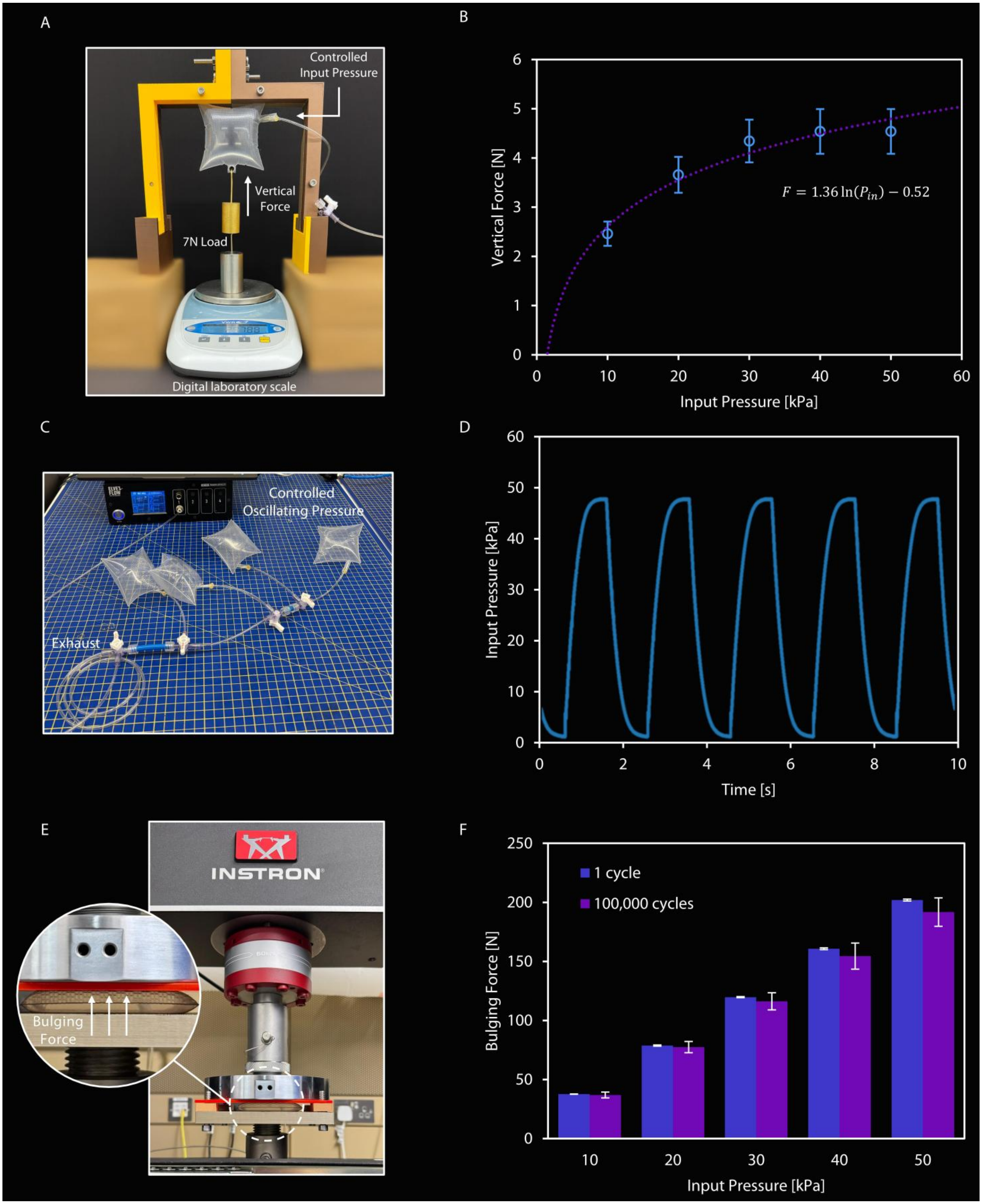
A
Controlled
Input Pressure
Vertical
Force
7N Load
Digital laboratory scale
B
Vertical Force [N]
Input Pressure [kPa]
$F = 1.36\ln(P_{in}) - 0.52$
C
Controlled
Oscillating Pressure
Exhaust
D
Input Pressure [kPa]
Time [s]
E
INSTRON
Bulging
Force
F
1 cycle
100,000 cycles
Bulging Force [N]
Input Pressure [kPa]

Figure S5: Force measurements and durability testing of vacuum–laser pouches. (A) Experimental setup for linear vertical force measurement of the pouches. **(B)** Mean vertical force and standard deviation of the pouches under different input pressures. **(C)** Experimental setup for the durability test of four pouches under controlled oscillating pressure. **(D)** Ten-second sample of the pressure oscillations generated by the microfluidic device. **(E)** Experimental setup for measuring the bulging force of the pouch using a tensile testing machine. **(F)** Comparison of new pouches and pouches subjected to 100,000 cycles of pressure oscillations, showing bulging force as a function of input pressure.

S6 Finite Element Method

We developed a fully automated finite element (FE) framework in Abaqus/CAE to simulate the deformation behavior of multi-cell bending actuators under internal pressure. The model generation, meshing, and analysis setup were scripted in Python using the Abaqus Scripting Interface, enabling systematic exploration of geometric and actuation parameters.

The script iterates through combinations of design parameters at a fixed applied pressure (pressures = [0.05] MPa), including the number of cells (n = [1, 2, 3]), and geometric ratios (x/l, y/l = 0.4-0.9). For each configuration, a 2D shell model representing a chain of connected rectangular cells is constructed. The internal geometry is defined by the parameters (*x/l*, *y/l*). At the same time, a seal width of 1 mm is maintained along the cell boundaries to replicate the thermoplastic welds.

The bending actuator is modeled as a bilayer shell structure with linear elastic-plastic material behavior. The elastic properties are defined as E = 350 MPa and ν = 0.47 (measured from tensile testing, see Section S2), and plastic hardening is described by a nine-point true stress-plastic strain curve (10.1-21.2 MPa, 0.00-0.37 strain). Each layer is assigned a Homogeneous Shell Section of 0.1 mm thickness. The two shell layers are tied together using an Abaqus Tie constraint to ensure perfect bonding between edge surfaces.

Triangular shell elements (S3) are used to mesh the geometry with an element size of 1 mm. A static, geometrically nonlinear step (Static, General, nlgeom = ON) is used to capture large deformations, with light automatic stabilization. Uniform pressure loads are applied to both inner surfaces of the pouch to simulate inflation. To eliminate rigid-body motion, an Encastre boundary condition is imposed at one corner vertex.

The automated framework generates a distinct analysis job for each parameter combination, with multithreading enabled (10 CPUs, 1 GPU). All simulations assume consistent SI-based units (mm, MPa).

This parametric FEM framework enables rapid generation and evaluation of hundreds of actuator geometries, providing a dataset of deformation responses used for surrogate model fitting (see Section S7).

S7 Surrogate Model Fitting

This section describes the model used to predict the bending angle θ as a function of geometric and design parameters (x/l, y/l, n), and the procedure used to fit it.

S7.1 Polynomial Regression

We assume that θ depends smoothly and nonlinearly on (x/l, y/l, n). To capture this dependence, we construct a quadratic polynomial surrogate with interaction terms. Let $z_1 = x/l$, $z_2 = y/l$, and $z_3 = n$.
The surrogate model takes the form

$$\theta(z_1, z_2, z_3) = \beta_0 + \beta_1 z_1 + \beta_2 z_2 + \beta_3 z_3 + \beta_4 z_1^2 + \beta_5 z_2^2 + \beta_6 z_3^2 + \beta_7 z_1 z_2 + \beta_8 z_1 z_3 + \beta_9 z_2 z_3 \tag{4}$$

where $\{\beta_i\}_{i=0}^{9}$ are regression coefficients to be determined.

The complete FEM dataset at a fixed pressure of 50 kPa (see Fig. 2F) is used for model fitting. After polynomial feature expansion, all features are normalised to a common scale before regression. Let **b** denote the vector of all bending angles obtained from FEM, and let **Φ** be the design matrix containing the expanded polynomial features of (x/l, y/l, n). The regression coefficients are then obtained by ordinary least squares:

$$\hat{\beta} = \underset{\beta}{\operatorname{argmin}} \ \| \mathbf{b} - \mathbf{\Phi}\beta \|_2^2 \tag{5}$$

computed numerically using a QR-based solver (Python library, scikit-learn [31]).

S7.2 Model Evaluation

We evaluate the model using the in-sample mean squared error (MSE) and coefficient of determination (R^2):

$$\mathrm{MSE} = \frac{1}{N}\sum_{i=1}^{N}(\theta_i - \hat{\theta}_i)^2, \qquad R^2 = 1 - \frac{\sum_{i=1}^{N}(\theta_i - \hat{\theta}_i)^2}{\sum_{i=1}^{N}(\theta_i - \bar{\theta})^2} \tag{6}$$

where θ_i are the FEM bending angles, $\hat{\theta}_i$ are the surrogate predictions, and $\bar{\theta}$ is their mean. In this study, the surrogate achieved an MSE of approximately 16.2 and an R^2 score of 0.95 (in-sample), indicating that the quadratic model captures the majority of the variance with an average prediction error of approximately 4° bending angle.

S8 Bending Actuators Durability

To evaluate the durability of bending actuators fabricated using the vacuum–laser method, we test four samples of a bending actuator with identical geometric features (x/l = 0.9, y/l = 0.4, n = 5). The

actuators are connected to a microfluidic pressure controller (OB1 MK4, Elveflow, see Fig. S6B) to generate oscillating pressure between 0 kPa and 50 kPa with a cycle period of 2 s (see Fig. S5D). We measure the bending angle of the four samples after 1, 10,000, 50,000, and 100,000 pressure cycles, and report the results in Fig. S6C. The results show less than a 5% reduction in bending angle after 100,000 cycles of oscillating pressure, decreasing from 179° at 1 cycle to 173° after 100,000 cycles, demonstrating the reliability of the bending actuators. These findings are consistent with the previous durability tests performed on square-shaped pouches, where bulging force measurements were used to assess performance degradation (see Fig. S5F).

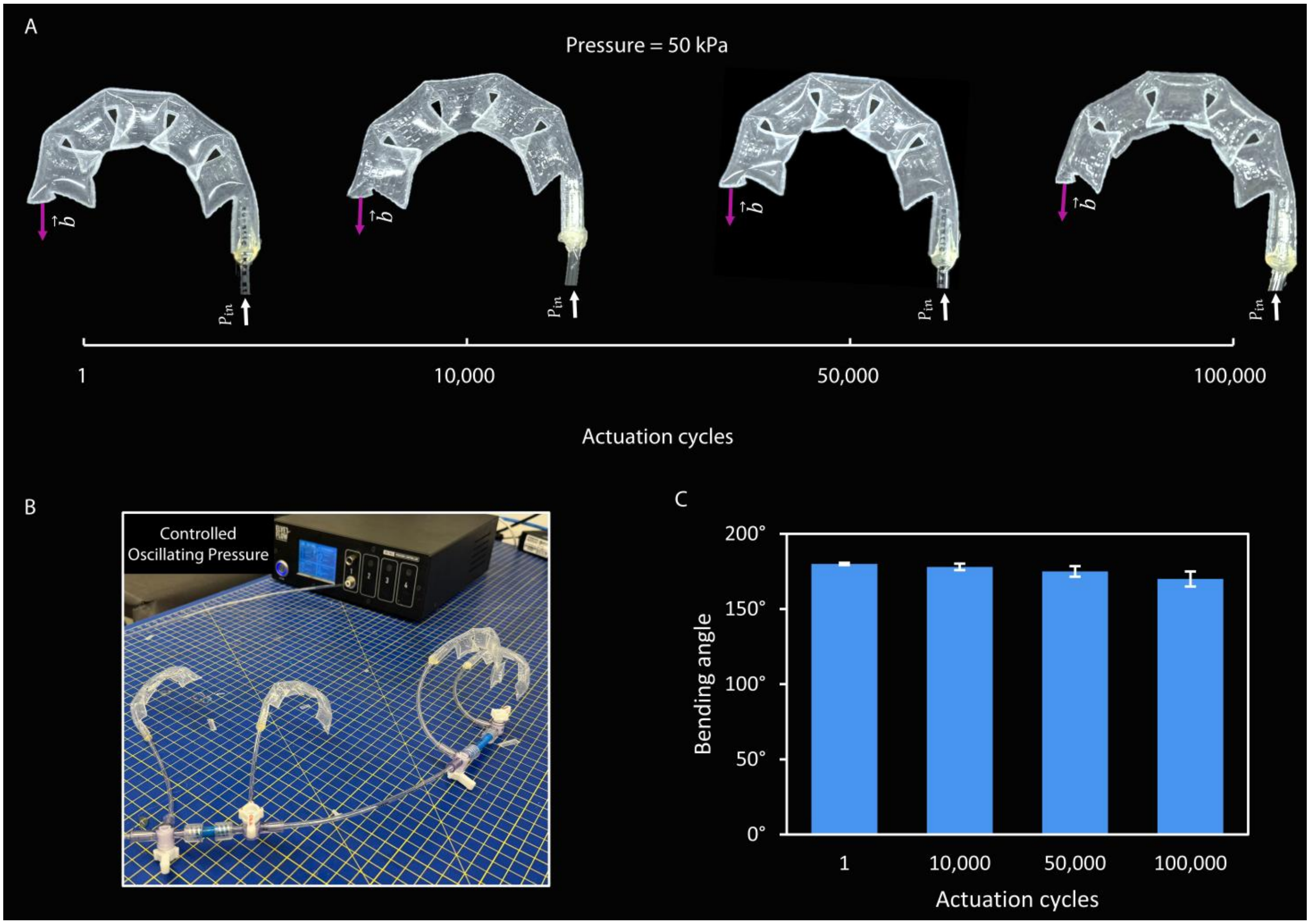

Figure S6: Durability test of the bending actuators. (A) Images of an inflated bending actuator at 50 kPa at different stages during oscillating pressure cycling. **(B)** Four bending actuators connected to the microfluidic device for durability testing. **(C)** Mean bending angle and standard deviation of the actuators measured at different stages of oscillating pressure cycling.

S9 Bending Actuators Scalability

Here, we explore the scalability of bending actuators fabricated using the vacuum–laser manufacturing technique within the geometric design space explored in this work. We fabricate seven samples of bending actuators with identical geometric features ($x/l = 0.9$, $y/l = 0.4$, $n = 5$), uniformly scaled in size by factors of [0.25, 0.5, 0.75, 1, 1.25, 1.5, 1.75, 2] (see Fig. S7A). All samples are tested by inflating them to an internal pressure of 50 kPa and recording the resulting bending angle (see Fig. S7B and C).

We identify a down-scaling limit at the ×0.5 scale, where the bending actuator fails to actuate due to excessively small internal channels that lead to immediate kinking at the actuator inlet. Based on this observation, the minimum viable inlet width is determined to be 6 mm, corresponding to the ×0.75 scaled actuator, which has a total width of 7.5 mm with seal widths of 0.75 mm on each side. In contrast, no limitation is observed during up-scaling: all actuators from the ×0.75 to the ×2 scale produce almost identical bending angles. Therefore, considering the identified down-scaling limit, the proposed manufacturing technique and design space support scalable fabrication of bending actuators within the range tested.

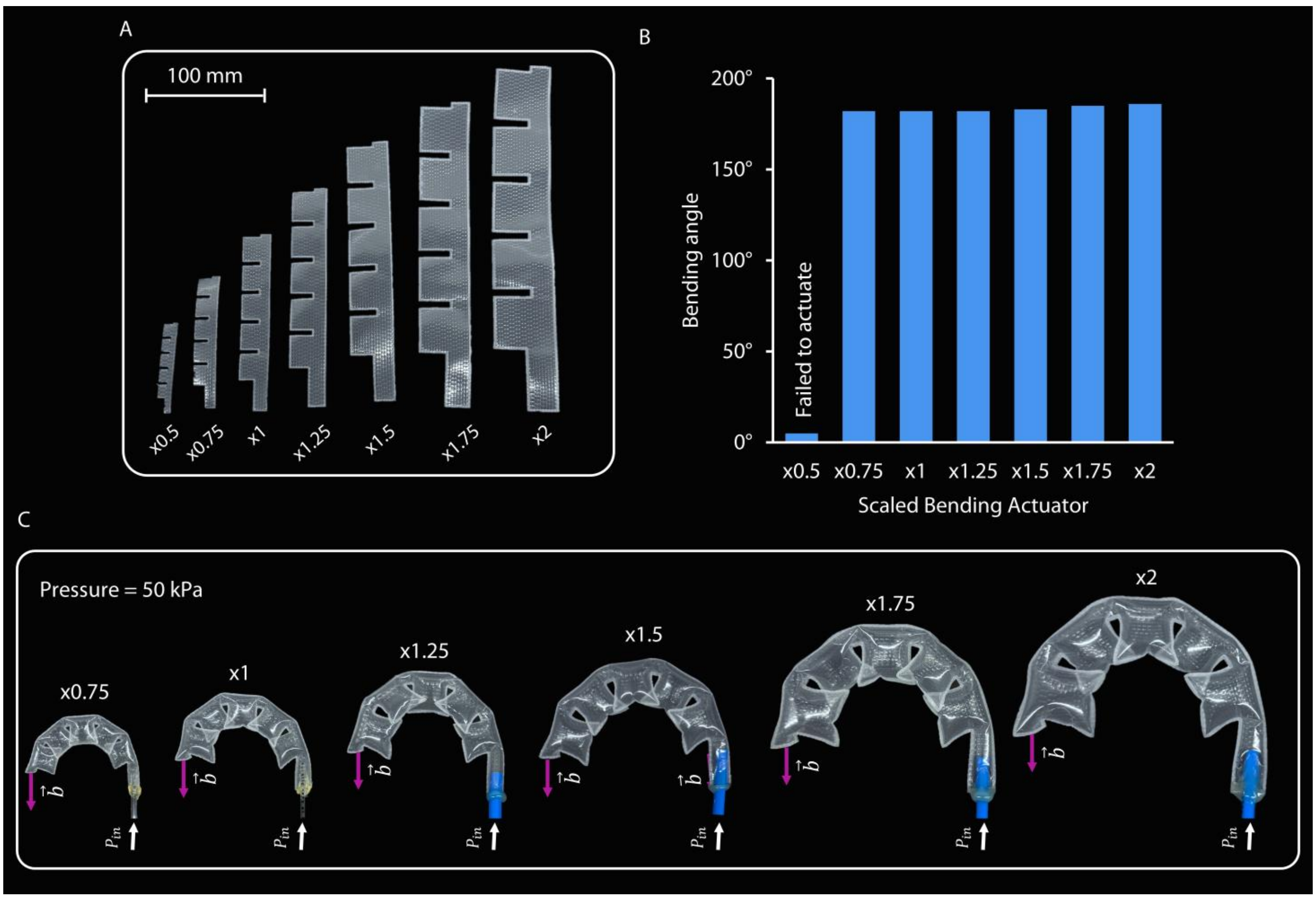

Figure S7: Scalability of the bending actuators. (A) Bending actuators scaled relative to the baseline design, including scale factors from ×0.25 to ×2. **(B)** Bending angle measurements upon inflation at 50 kPa, showing failure of the ×0.5 actuator and indicating the down-scaling limit. **(C)** Images of inflated bending actuators from the ×0.75 to ×2 scaled versions.

S10 Out-of-Plane Bending with Stacked Bending Actuators

To explore multi-degree-of-freedom bending using our bending actuators, we investigate how selective actuation of a stacked bending actuator influences the bending orientation. The stacked configuration consists of three bending actuator layers ($n_S = 3$). For simplicity, only the two outer actuators are actively pressurized, while the middle actuator remains uninflated. We test three possible actuation combinations of the two outer actuators: [ON, OFF], [OFF, ON], and [ON, ON].

We observe that selective actuation enables bending in three distinct directions, including two out-of-plane bending modes (Fig. S8A and B) relative to the original in-plane bending direction (Fig. S8C). Notably, when only one of the outer bending actuators is inflated, the entire stacked actuator bends toward the inflated side due to the increased stiffness induced by pressurization on that side.

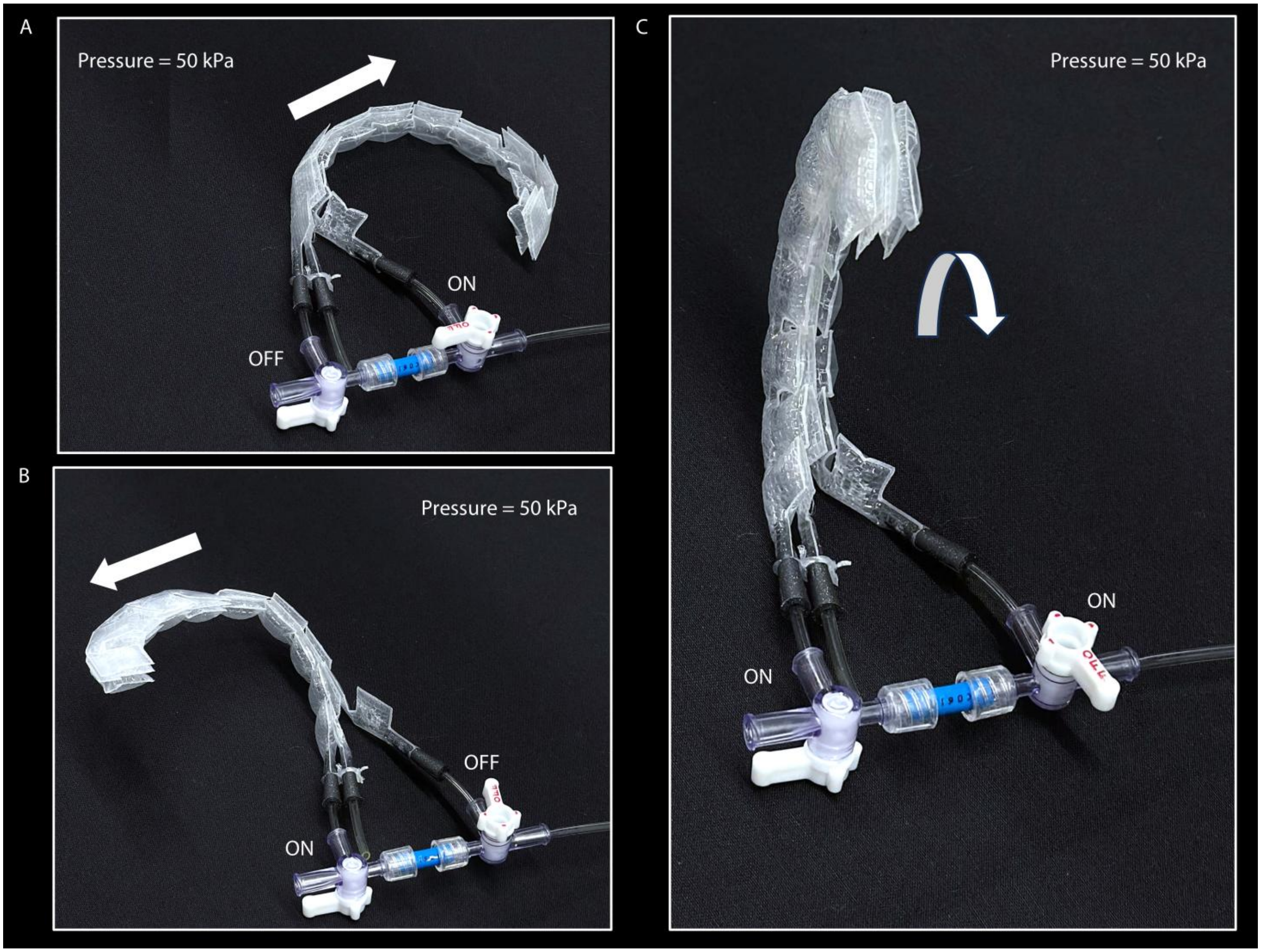

Figure S8: Out-of-plane bending enabled by stacked bending actuators. (A) Out-of-plane bending achieved by actuating only the rightmost bending actuator. **(B)** Out-of-plane bending achieved by actuating only the leftmost bending actuator. **(C)** In-plane bending obtained when both outer bending actuators are actuated simultaneously.

S11 Robustness of the Robotic Gripper and Gripping Force

To evaluate the robustness of our robotic gripper, we fabricate three commonly encountered target objects—a cylinder, a cube, and a sphere—using a 3D printer (Bambu Lab X1 Carbon). Each object fits within an 80 mm by 80 mm by 80 mm bounding box (Fig. S9A). We assess the picking success rate by consecutively attempting to grasp each object 20 times. The gripper demonstrates acceptable performance across all targets (Fig. S9B and C). Notably, the cylindrical object is successfully picked in all trials, while the cube and sphere exhibit success rates of 90% and 75%, respectively. Failures are primarily caused by slipping during grasping. These results further validate the 100% success rate observed when grasping real-life objects shown in Fig. 4D, as those objects possess semi-cylindrical geometries that are particularly well suited for our gripper design.

Additionally, we measure the maximum gripping force of the robotic gripper using a tensile testing machine (Instron). The gripper is fixed to the bottom platform of the machine and connected to the load cell using an inextensible, non-flexible strap. As the strap is pulled upward, the gripping force increases and reaches a peak when the strap comes into contact with the tips of the bending actuators, at approximately 4 N. Beyond this point, the strap progressively slips out of the gripper, causing the measured force to decrease to 0 N (Fig. S9D; see insets for images captured at different stages of the test). Therefore, the maximum gripping force generated by our robotic gripper is approximately 4 N.

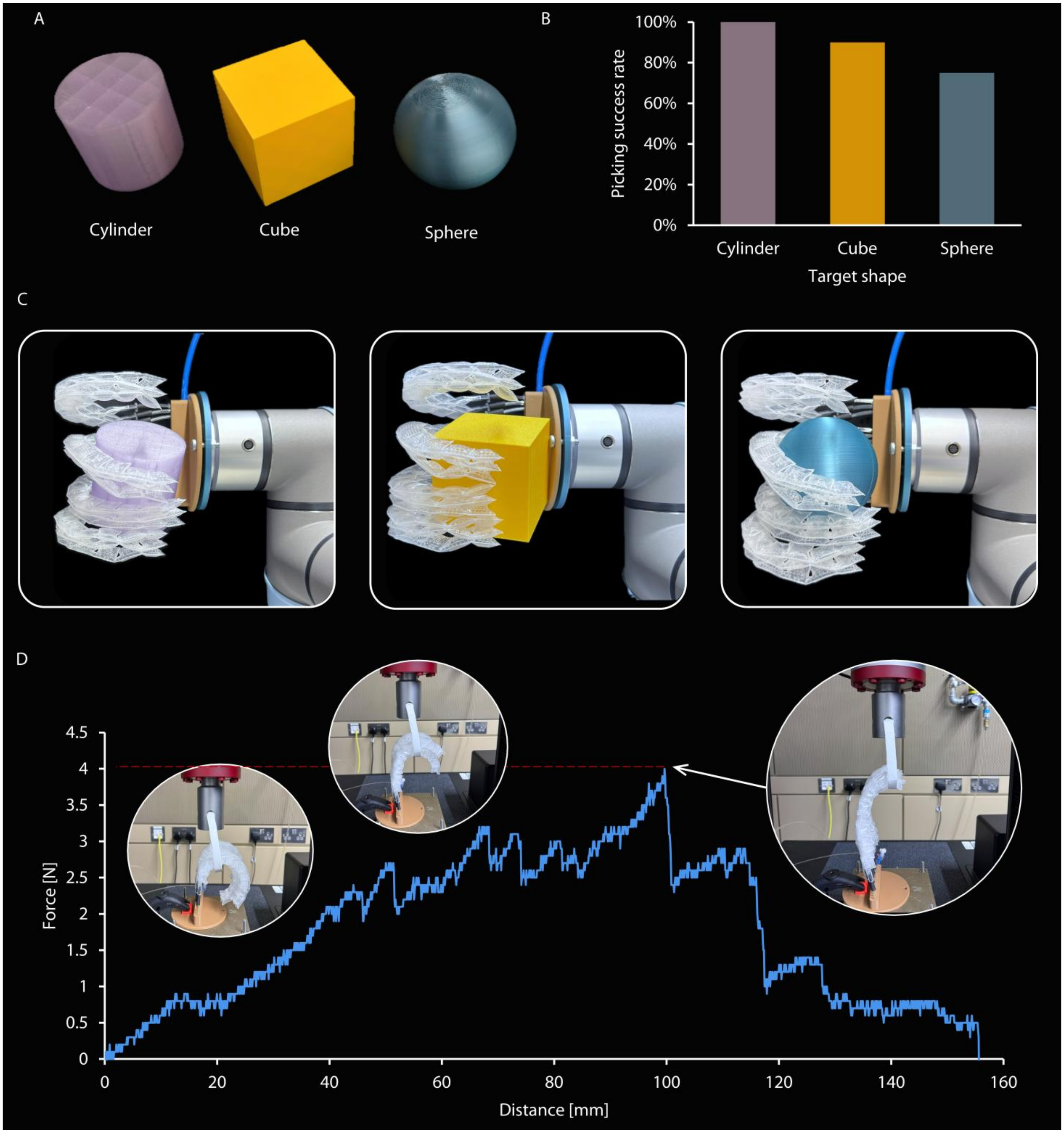

Figure S9: Robustness and gripping force characterization of the robotic gripper. (A) 3D-printed target objects (cylinder, cube, and sphere) used for grasping tests. **(B)** Success rates for grasping different target objects over 20 consecutive trials. **(C)** Images demonstrating successful grasping of the three target shapes. **(D)** Gripping force measurement using a tensile testing machine, with insets showing different stages of the test.

S12 Crawler and Swimmer Design Sketches and Locomotion Characterization

The geometric designs of the crawler and swimmer are shown in Fig. S10A and B, respectively. Both robots are fabricated using the same vacuum–laser manufacturing process described in the main text, with a seal width of 1 mm. The crawler consists of two multi-cell legs arranged with a fixed angular offset to promote asymmetric frictional interaction with the ground, while the swimmer replaces the last unit cells with enlarged paddle-like structures to enhance fluid interaction.

To quantitatively characterize locomotion performance, we perform a systematic study of the effect of actuation frequency on locomotion speed while keeping the input pressure fixed at 50 kPa. For both the crawler and the swimmer, pressure oscillations are generated using a microfluidic pressure controller (OB1 MK4, Elveflow), and the actuation frequency is varied while maintaining identical pressure amplitude. Locomotion speed is measured from video recordings (DJI Action 4, 1080p, 240 fps) and normalized by body length, and the resulting speeds are reported in body lengths per second (BL/s).

Figure S10C shows the measured locomotion speed of the crawler as a function of actuation frequency. The crawler exhibits a clear frequency-dependent response, with negligible motion at low frequencies, a peak speed of approximately 0.8 BL/s at 1.8 Hz, and reduced performance at higher frequencies, likely due to incomplete deformation recovery within each cycle.

Similarly, Fig. S10D reports the frequency-dependent locomotion speed of the swimmer. The swimmer reaches a maximum speed of approximately 0.5 BL/s at an actuation frequency around 1.2 Hz, followed by a gradual decline in speed at higher frequencies.

These results demonstrate that, under fixed pressure conditions, locomotion performance for both crawler and swimmer is strongly governed by actuation frequency, highlighting the importance of dynamic tuning for achieving effective terrestrial and aquatic locomotion.

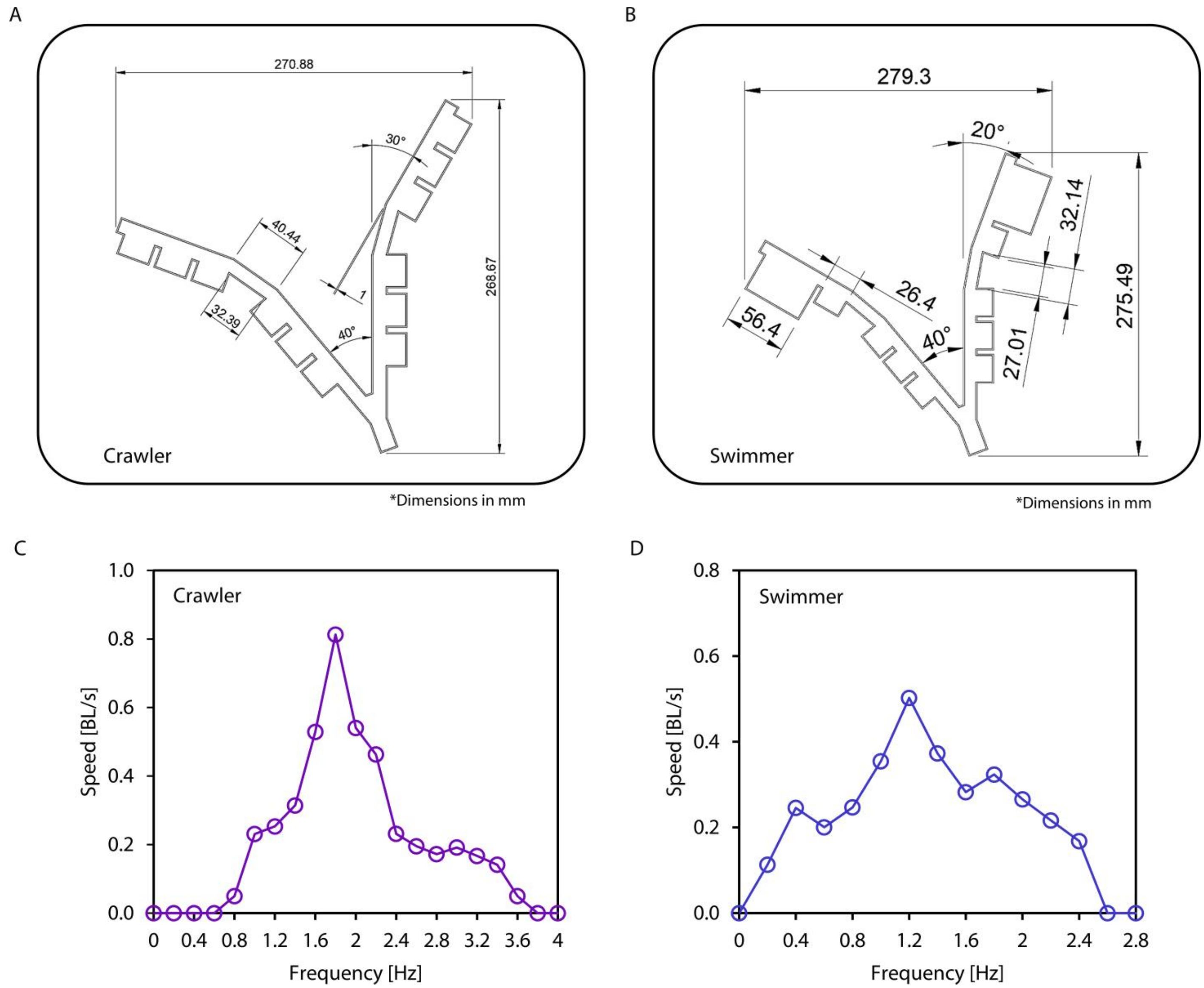

Figure S10: Crawler and swimmer design and locomotion characterization. (A) The crawler design sketch with 1 mm edges for sealing. **(B)** The swimmer design sketch with 1 mm edges for sealing. **(C)** Locomotion speed of the crawler as a function of actuation frequency at a fixed input pressure of 50 kPa, reported in body lengths per second (BL/s). **(D)** Locomotion speed of the swimmer as a function of actuation frequency at a fixed input pressure of 50 kPa, reported in body lengths per second (BL/s).

S13 Force Output and Bursting Pressure of Silicone Pouches

To compare the performance of vacuum–laser thermoplastic pouches with a commonly used class of materials in soft robotics, we fabricate silicone-based pouches using Ecoflex 30 (Smooth-On; Fig. S11A). We note that a wide range of silicone elastomers with different mechanical properties is available; however, as this comparison is intended as a supplementary benchmark rather than an exhaustive materials study, we focus here on Ecoflex 30 as one of the most commonly used silicone rubbers in soft robotics. Due to the manual nature of silicone processing, producing sheets thinner than 0.2 mm proved challenging. After multiple trials, uniform silicone sheets were obtained by pouring the liquid mixture onto a flat acrylic substrate and placing a second acrylic plate (5 mm thickness) on top as a press during curing (4 h curing time).

Once cured, silicone sheets were cut into 140 mm × 240 mm rectangles, folded, and sealed along three edges using additional uncured silicone as an adhesive, resulting in 100 mm × 100 mm silicone pouches. A seal width of 20 mm was intentionally used to ensure reliable bonding, which is substantially larger than the 1 mm seal width used in vacuum–laser pouches. This difference reflects the limitations of manual fabrication compared to laser-based sealing. Overall, the fabrication time for a silicone pouch was approximately 8 h; even when using fast-curing silicones (30 min cure), the total fabrication time remains on the order of 1 h, which is significantly longer than the < 10 min required to fabricate a vacuum–laser pouch of identical size.

We evaluate the bulging force performance of silicone pouches using the same experimental protocol described in Section S5 (Supporting Information), employing a tensile testing machine (Instron; Fig. S11B). The internal pressure is increased incrementally from 1 kPa in 1 kPa steps. Silicone pouches rupture at an internal pressure of approximately 4 kPa, producing a maximum average bulging force of 27.8 N (Fig. S11C).

Notably, silicone pouches exhibit a substantially lower bursting pressure than vacuum–laser thermoplastic pouches of comparable thickness, which sustain pressures up to ∼70 kPa (see Section S3, Supporting Information). Consequently, to operate silicone pouches at higher pressures, a significant increase in material thickness would be required, whereas thin silicone pouches remain limited to a narrow operating range (0–4 kPa). In contrast, vacuum–laser pouches achieve bulging forces of approximately 202 N at 50 kPa (see Section S5, Supporting Information).

Due to the highly extensible nature of silicone elastomers, the silicone pouch undergoes pronounced areal expansion during inflation, resulting in a substantially larger contact area with the top compression plate of approximately 14,061 mm^2 (118.5 mm × 118.5 mm). Using this contact area, we quantify the normalized bulging force by dividing the measured force by the effective contact area, following the same definition as in Section S5 (Supporting Information). The resulting normalized bulging force is approximately 0.002 N/mm^2 at an input pressure of 4 kPa. This value is about 25% lower than the normalized bulging force of approximately 0.0025 N/mm^2 measured at the same pressure for thermoplastic pouches fabricated using the vacuum–laser technique (see Fig. S11D). This reduced output force is attributed to increased energy dissipation, in particular strain energy associated with the large elastic deformations inherent to silicone materials.

Together, these results highlight the advantages of vacuum–laser-fabricated thermoplastic pouches in terms of fabrication speed, pressure tolerance, force output, and reduced strain energy losses, while providing a quantitative comparison with silicone-based soft actuators fabricated using conventional manual techniques.

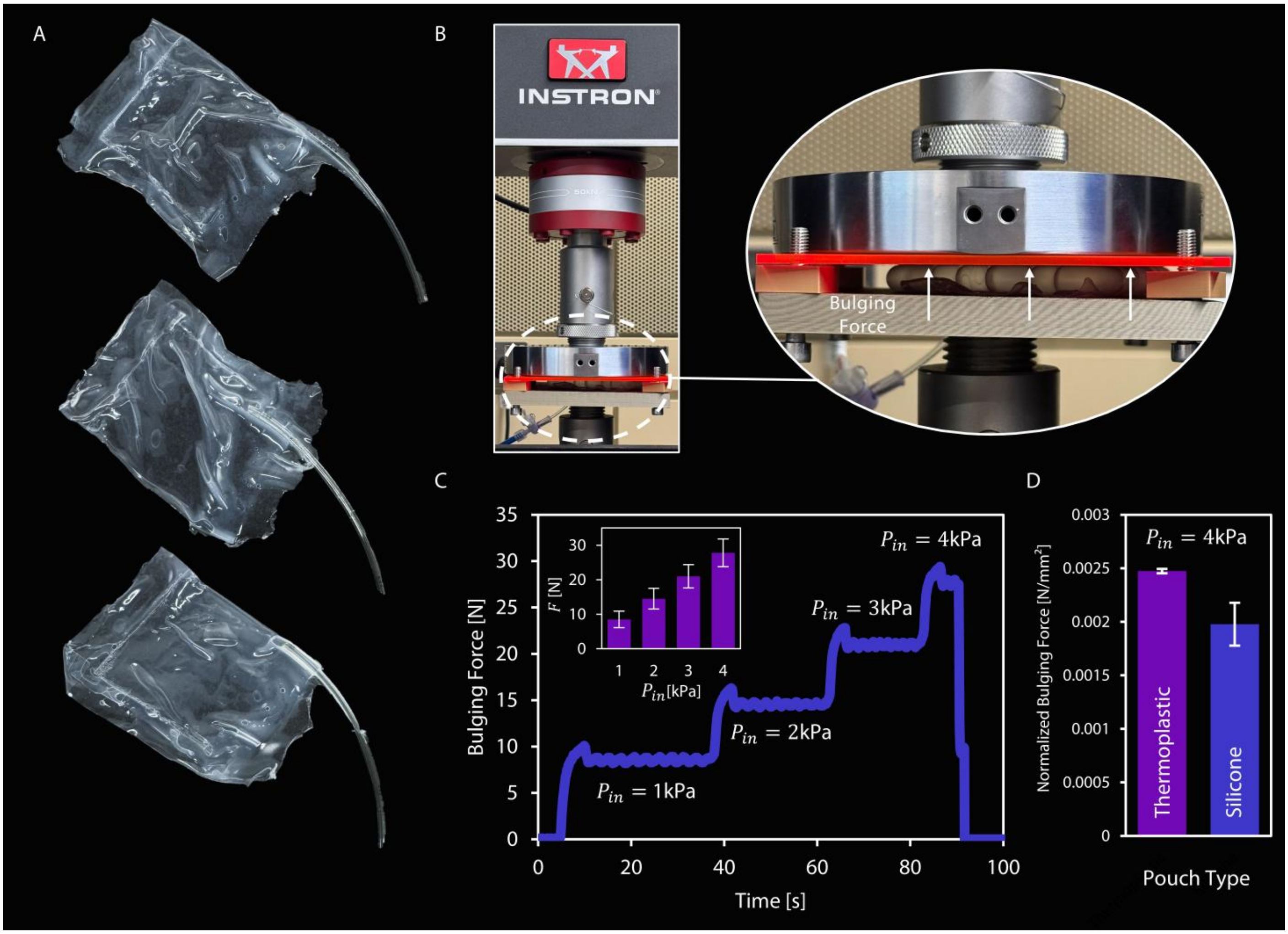

Figure S11: Force output and bursting behavior of silicone pouches. (A) Fabricated silicone pouches (Ecoflex 30) prior to testing. **(B)** Experimental setup for bulging force measurements using a tensile testing machine (Instron), with a close-up view illustrating the bulging deformation and the direction of the measured force. **(C)** Bulging force measured by the Instron as a function of time while the internal pressure is increased stepwise from 1 to 4 kPa, showing stable force plateaus at each pressure level and sudden rupture at 4 kPa. The inset shows the mean and standard deviation of the maximum bulging force at each pressure level, obtained from three independently fabricated silicone pouches. **(D)** Comparison of normalized bulging force between silicone pouches and thermoplastic pouches fabricated using the vacuum–laser technique at an input pressure of 4 kPa. Bars represent the mean value, and error bars indicate the standard deviation across samples.

Reference

[31] Fabian Pedregosa et al. “Scikit-learn: Machine Learning in Python”. In: Journal of Machine Learning Research 12 (2011), pp. 2825-2830.
